# Metaheuristic Approach to Synthesis of Suspension System of Mobile Robot for Mining Infrastructure Inspection

**DOI:** 10.3390/s22228839

**Published:** 2022-11-15

**Authors:** Mateusz Malarczyk, Marcin Kaminski, Jaroslaw Szrek

**Affiliations:** 1 Department of Electrical Machines, Drives and Measurements, Faculty of Electrical Engineering, Wroclaw University of Science and Technology, Smoluchowskiego 19, 50-372 Wroclaw, Poland; 2 Department of Fundamentals of Machine Design and Mechatronic Systems, Faculty of Mechanical Engineering, Wroclaw University of Science and Technology, Lukasiewicza 7/9, 50-372 Wroclaw, Poland

**Keywords:** geometrical synthesis, four-bar mechanism, Chameleon Swarm Algorithm, wheel-legged robot, optimization

## Abstract

The article describes the problem of geometric synthesis of the inspection robot suspension system, designed for operation in difficult conditions with the presence of scattered obstacles. The exemplary application of a mine infrastructure inspection robot is developed and supported by the ideas. The brief introduction presents current trends, requirements and known design approaches of platforms enabled to cross the obstacles. The idea of a nature-inspired wheel-legged robot is given, and the general outline of its characteristics is provided. Then the general idea of kinematic system elements selection is discussed. The main subject of geometrical synthesis of the chosen four-bar mechanism is described in detail. The mathematical model of the suspension and connections between the parts of the structure is clarified. The well-known analytical approach of brute force search is analyzed and validated. Then the method inspired by the branch and bound algorithm is developed. Finally, a novel application of the nature-inspired algorithm (the Chameleon Swarm Algorithm) to synthesis is proposed. The obtained results are analyzed, and a brief comparison of methods is given. The successful implementation of the algorithm is presented. The obtained results are effectively tested with simulations and experimental tests. The designed structure developed with the CSA is assembled and attached to the prototype of a 14-DOF wheel-legged robot. Furthermore, the principles of walking and the elements forming the control structure were also discussed. The paper is summarized with the description of the developed wheel-legged robot LegVan 1v2.

## 1. Introduction

Autonomous platforms and robots have become more popular in recent years. The variety of applications is constantly growing. The driving environment determines the construction of the platform [1,2,3,4,5]. In order to ensure the possibility of operating in industrial or mining areas and serve as in-building delivery systems and cooperate with humans in traffic, it is necessary for a robot to traverse obstacles such as rocks, curbs and unevenness. Additionally, the robot should be leveled during the operation with sensory equipment on uneven terrain [1,2].

Different suspension designs are known, including active dampers, multi-link suspension or hydro-pneumatic self-levelling systems developed in 1950s. The different designs are also characterized with different qualities. While hydro-pneumatic suspensions provide good damping characteristics and give the possibility to control the clearance, they require pumps and oil containers and are vulnerable to damage [6,7,8]. The other mentioned systems and technologies also require a complex mechanical structure which is equipped with drives and sensors. The systems require time-consuming design in order to ensure efficiency and reliability [9].

**Figure 1:**
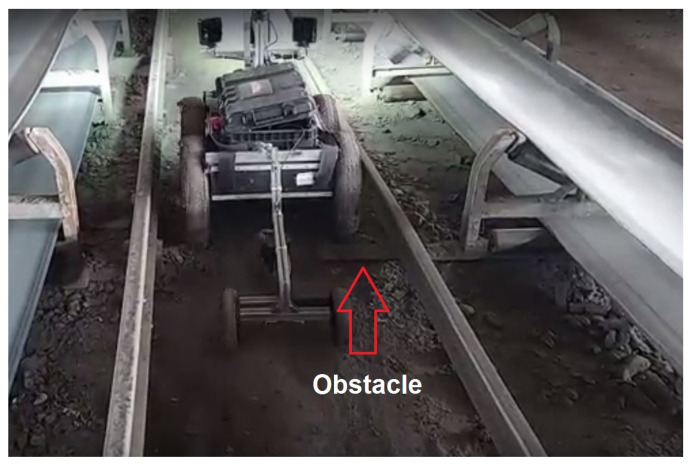
The robot driving alongside the mine transportation machinery and inspecting a sample mine infrastructure—the belt conveyor.

The robotic platforms in modern underground mines can easily interact with the environment when any obstacle (such as rocks, mud, rails, cable entries, supports and other mine infrastructure; see Figure 1) is present in the narrow aisles, the movement of the robots may be disrupted [10,11]. The stability and levelness of the robots are particularly important during sensory data recording [12]. The requirement of stable movement is especially crucial when inspecting the long segments of belt conveyors.

There is usually no possibility of driving next to the obstacle, so the only possibility is to wait for the maintenance workers or systems to remove the problem. Such a problem generally does not exist in an urban environment, but other elements may block the movement of robotic platforms, such as rocks, curbs or steep slopes. Living creatures are resistant to the mentioned problems. The possibility of walking introduces an additional degree of freedom instead of the movement in “flat” driving. This led to the design of a four-legged robot which drives in most situations and can walk over obstacles in special cases.

**Figure 2:**
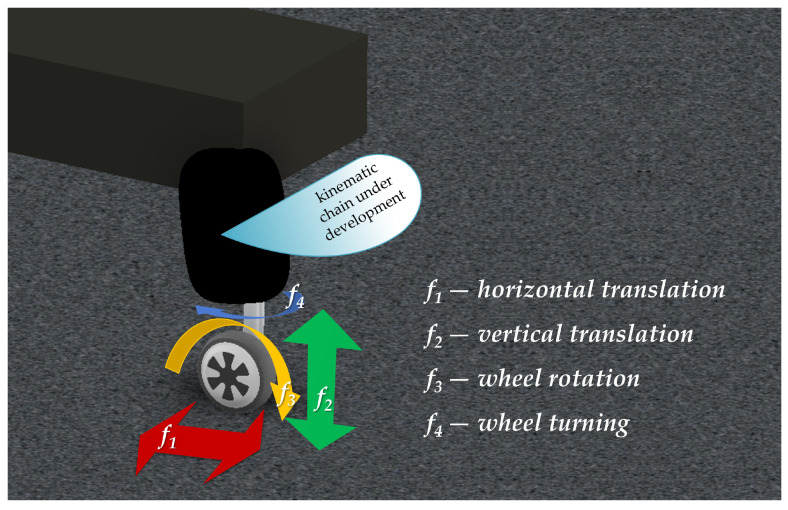
The front leg of the robot—degrees of freedom.

Every leg is equipped with a brushless DC motor at the end. The motor is responsible for propelling the platform into driving mode. Additionally, two more drives are attached to every limb. The first one, the linear actuator, is needed to move the wheel in a horizontal plane. The second one is responsible for the movement in the vertical plane. In order to ensure the steering of the platform, two limbs are equipped with motors that enable turning the wheels around the axis perpendicular to the driving surface (Figure 2). The total number of degrees of freedom, and thus the required number of drives, equals 14. The front legs are equipped with four drives, and the rear ones incorporate only three drives. In order to reduce connections and the complexity of the suspension four-bar mechanism is developed.

Real industrial or scientific issues can often be characterized as complex, with an uncertainty of parameters, time-variant, with non-linear dependencies of state variables, etc. On the other side, the selection of parameter values is a significant issue. According to mentioned properties of the objects, it can be a difficult challenge. For this purpose, the growing interests of scientists and engineers are focused on Swarm Inspired algorithms. Searching for the best solution can be considered a change in the population. Several modifications, commonly based on basic calculations, are performed in the subsequent iteration. Updates of individuals are achieved using the values of the initially defined cost function, and it also includes local interactions between elements. Transformation of the group leads to the best values of the parameters needed in the problem [13,14,15]. The relationship between the subject of the optimization and the algorithm was established based on the error value (cost/objective function). It enables the simple and universal application of the algorithm for different tasks. It should be noticed that the optimizer uses only selected real values. Detailed recalculations with complex equations describing the problem are not necessary. Another advantage is related to the possibility of searching for a solution that takes into account many assumptions and limitations of the obtained values. Concluding, simplicity of implementation, efficiency and a wide range of available algorithms have resulted in the growing use of SI algorithms. A typical example application of the Artificial Bee Colony for decision-making problems in the industrial system with an arm robot can be shown [16]. Another application, often described in journals or conference proceedings, is tuning of the state controller gains (applied for electrical drives with PMSM machines or systems with elastic couplings) [17,18]. Not only calculations but objects as well can be optimized. The Cuckoo Search algorithm was used in the design process of the brushless DC machine (the goal was torque ripple reduction) [19]. The interdisciplinary nature and diversity of the fields in which the SI algorithms are used illustrate the possibility of training the neural network (without gradient calculation) with the Particle Swarm Optimization and analysis in biochemistry [20,21]. Due to the above considerations, the Chameleon Swarm Algorithm, in this work, was chosen to optimize the mechanical construction of the wheel-legged inspection robot. Moreover, the selection of the Chameleon Swarm Algorithm was also based on the literature analysis. The results of the study provided by Braik [22] were taken into account when the recently available algorithms were considered.

The paper is divided into six sections providing the subsequent parts of the problem solution successively. The introduction shows fundamentals and general ideas of the presented research. Then, the synthesis task is described in detail, and the mathematical model of the suspension structure is established. In the third section, the standard methods of calculating the dimensions are briefly described, and the exemplary results are presented. The improvement of the very basic brute-force algorithm is suggested, and the advantages and disadvantages are discussed. In the following sections, the novel metaheuristic algorithm is introduced with a detailed description of the computation fundamentals and the next steps of the process. The section also provides simulation results and proves the initial assumption that the optimization algorithm can be applied to the geometrical synthesis of the suspension structure. The simulation results are supported by the experimental results presented in the next section of the paper. Not only the structure of the suspension system is presented. Moreover, the sensors, the control system and the drives are included in the description. Finally, the conclusions are drawn, and further research plans are presented.

## 2. Problem Formulation, Type Synthesis and Desired Wheel Trajectory

The problem of robot suspension synthesis is a complex computation of segment lengths, fixing point positions and defining angular constraints between the parts. The process aims to reproduce the requested trajectory and provide linear relation between propelling drive, and the displacement [23,24,25,26,27,28].

**Figure 3:**
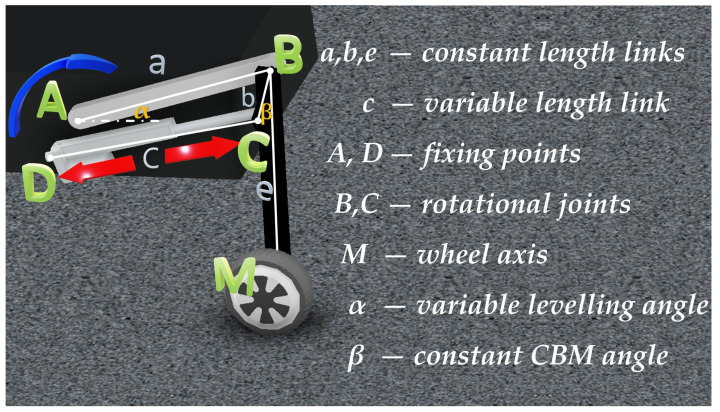
Four-bar linkage structure with reference motion trajectory.

In the described design, it was defined to use only one drive for platform levelling. The second motor was responsible for the walking task (horizontal wheel displacement). The required four degrees of freedom

(1) $$\sum f_{i}=4$$

are calculated for the turnable limb. One of the degrees of freedom may be neglected in structural analysis because this ability is achieved by attaching the DC motor with a gear along the bar. Moreover, the DOF connected with wheel propelling may be disregarded, as the required trajectory is calculated for the wheel axis. Taking into account these assumptions, the theoretical mobility (number of drives) equals 2. The intermediate chain structure may be developed using the structural analysis methods [29]. In order to mount the drives on the platform, the four-bar linkage structure was chosen (Figure 3).

The mounting points were A and D. The levelling motor was attached as a rotary actuator at point A. The movement of the rocker AB (which length *a* is constant) is transferred to BC bar which is characterized with length *b*. The angle between BC bar and BM link (β) is constant. BM is the part of the length *e* connecting the joint B with the wheel’s axis. The desired trajectory equals the movement of point M. The four-bar structure is also attached to the platform in point D, and the length of CD linkage (*c*) is customizable. This was achieved by mounting a linear actuator. The designed mobility and the required drives were indicated by the arrows in Figure 3.

The missing lengths, angles and attachment points are calculated during geometrical synthesis. The desired trajectory was linear, and the second goal was to achieve linear relation between bar AB’s angular displacement and point M’s vertical displacement. The structure should also give the possibility to attach drives to the platform and, finally, should be compact.

Direct calculation of the missing sizes is impossible. However, making some presumptions and defining quality indicators helps to solve the problem. For the research purpose, the quality was measured as an error between the desired position of point M on the trajectory and the computed one. It was defined in a two-dimensional environment. The equation defining the quality indicator is given below (2).

(2) $$w_{j}=s_{x}\sum_{i=1}^{n}x_{i}^{2}+s_{y}\sum_{i=1}^{n}y_{i}^{2}$$

where $s_{x},s_{y}$ are the weight coefficients and $x_{i},y_{i}$ are the distances between desired and computed position in *i*-th step. The coefficients have to be chosen with regard to the below relation (3):

(3) $$s_{x}>=0;\;s_{y}>=0\;and\;s_{x}+s_{y}=1.$$

This leads to the final definition of quality indicator (4):

(4) $$w_{j}=s_{x}\sum_{i=1}^{n}x_{i}^{2}+\left(1-s_{x}\right)\sum_{i=1}^{n}y_{i}^{2}.$$

The input for the synthesis is fixed position of joint A. At the beginning of the synthesis, only the extreme positions (the initial point $M_{i}$ and the final position $M_{f}$ are taken into account. Basing on that points rocker AB placement is computed and using Equation (5) lengths of AB and BM are calculated.

(5) $$\left\{ \begin{array}{c} e^{2}={\left(x_{B_{i}}-x_{M_{i}}\right)}^{2}-{\left(y_{B_{i}}-y_{M_{i}}\right)}^{2}\\ e^{2}={\left(x_{B_{f}}-x_{M_{f}}\right)}^{2}-{\left(y_{B_{f}}-y_{M_{f}}\right)}^{2} \end{array}\right.$$

where: *x, y* are the coordinates of point B and extreme position of trajectory for initial and final position respectively calculated from the following geometric relations (6):

(6) $$\begin{array}{c} x_{B_{i}}=a\cos\alpha_{i}\\ y_{B_{i}}=a\sin\alpha_{i}\\ x_{B_{f}}=a\cos\left(\alpha_{i}-\Delta \alpha \right)\\ y_{B_{f}}=a\sin\left(\alpha_{i}-\Delta \alpha \right). \end{array}$$

If the *a* and the *e* are known, the linkage mechanism is checked against the configuration where the movement of the bars is impossible because of mutual blocking. This may occur when points A, $B_{f}$ and $M_{f}$ are collinear. Moreover, both initial and final configuration point B has to be placed on the same side of the AM segment. This may be verified with Equation (7).

(7) $$\gamma_{allowed}+\alpha_{i}-\Delta \alpha <=atan2\left(y_{M_{f}},x_{M_{f}}\right).$$

In the next step, the range of point D coordinates is defined. The fastening point D must be placed on the platform construction and must not result in the “dead position” configuration. A minimal length of BC bar has to be given. Based on these parameters, the DC linkage length may be calculated based on Equation (8).

(8) $$\left\{ \begin{array}{c} B_{i}D\cos\Phi_{DB}-c\cos\Phi_{2_{i}}-\delta -b\cos\Phi_{2_{i}}=0\\ B_{i}D\sin\Phi_{DB}-c\sin\Phi_{2_{i}}-\delta -b\sin\Phi_{2_{i}}=0 \end{array}\right.$$

where: $\Phi_{DB}$ describes the angle between element DB and platform level; $\Phi_{2_{i}}$ is the angle between linkage BC and the level, when the B is initial position, δ is the angle between rocker DC and BC in initial position. The final part of the geometrical synthesis is calculation of the β angle.This is proceeded according to Equation (9). This is the last parameter that is calculated.

(9) $$\beta =atan2\left(y_{M_{i}}-y_{B_{i}},x_{M_{i}}-x_{B_{i}}\right)-\Phi_{2_{i}}.$$

After the process, all the suspension dimensions are known, and the simulation can be conducted. For research purposes, the simulation of only one drive was considered. The linear actuator (linkage DC) remains switched off at the required length. The only controlled drive is the one responsible for the angular movement of rocker AB around an axis in point A. The α angle is considered within the range defined by the desired $M_{i}$ and $M_{f}$ positions. The angular range is divided into equal steps, and intermediate positions are computed. The quality indicator (according to Equation (4)) is then calculated.

## 3. Non-Metaheuristic Methods

### 3.1. Brute-Force Search

The most commonly used method of finding the best solution for the given synthesis is brute-force search. It is based on the mathematical description given in the previous chapter. The main idea of such an approach is to define initial constraints and assume a search range for every calculated dimension.

**Figure 4:**
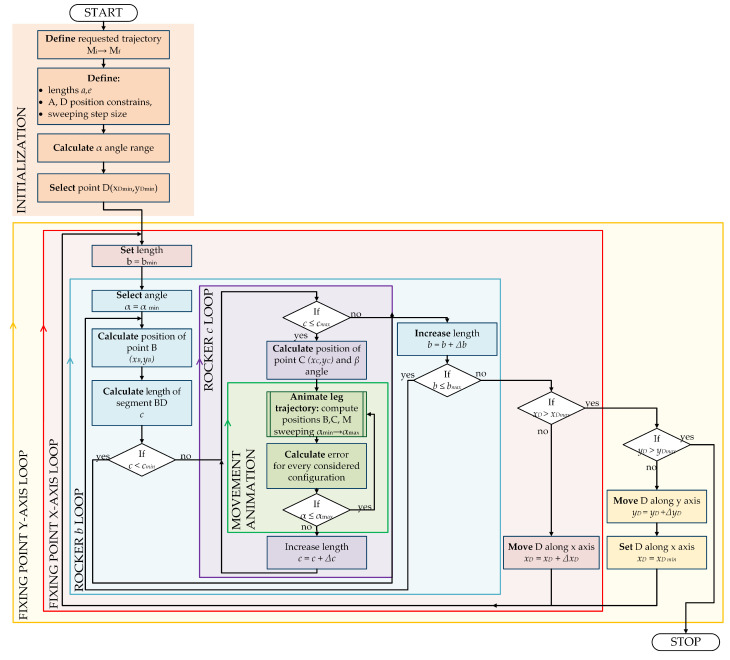
Flowchart of geometrical synthesis with the brute-force search method.

The process starts with values equal to lower bounds, and in every calculation, only one parameter is increased by a defined step size [30,31]. This leads to multiple loop-based search (Figure 4).

**Figure 5:**
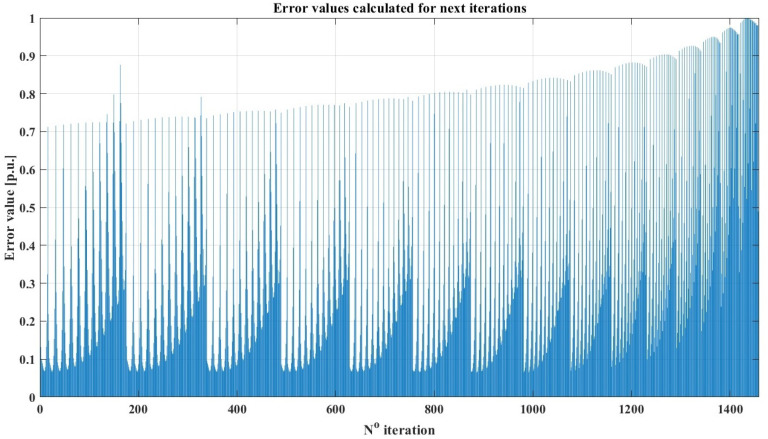
Error values in the subsequent iterations of the brute-force search method.

In the given problem, the main loop is responsible for sweeping the area for fixing point D. As there are two coordinates of the D position, in fact, the main loop consists of two sub-loops driven along x and y axes. Input values for the algorithm, which have to be predefined are: length of rocker AB (*a*) and rocker BM (*e*). The first nested loop is responsible for sweeping the range of element BC *b* length. The position of joint B is determined based on the initial position of the suspension system ($\alpha_{min}$). Based on that data, the length of the rocker CD (*c*) can be calculated. In order to reduce the number of iterations, it was decided to check if the obtained *c* is not too short (as the linear actuator represents it). If the solution is not rejected, the position of point C is calculated, and β is determined. The nested loop increases the length of *c* with a defined step. For every iteration, the external loop simulates the movement of the suspension system. The recurrence can be easily observed in the chart presenting the relative value of the quality indicator for the next iterations of the brute-force method (Figure 5). The figure presents the main disadvantage of the method: the error value in the last iterations of the process is significantly higher in comparison to the ones obtained in the initial steps of the process.

The described method allows for finding the draft of the system dimensions. The resolution of the search is limited with step size for every dimension (e.g., $\Delta_{c},\Delta_{b}$). The smaller the size is, the more iterations of the brute-force algorithm are required. As seen in the flowchart (Figure 4) and the given description, there is no feedback from the quality indicator to the search algorithm. This means every case is calculated, even if no improvement in trajectory is obtained. As every iteration requires a long time to compute and the results must be stored, the method is time and memory inefficient. Because of the mentioned disadvantages, such a method is suggested only for preliminary synthesis with very coarse resolution (for example, with a step size of 1 cm).

For the research, the brute-force search was implemented. The input values, search ranges and steps are gathered in Table 1.

### 3.2. Branch and Bound Inspired Algorithm

**Figure 6:**
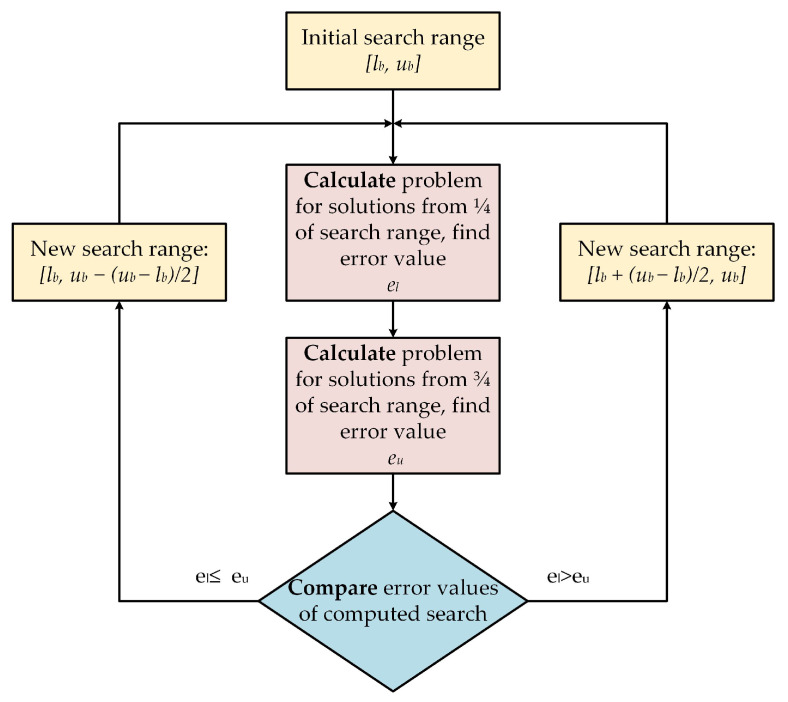
The idea of rejecting improper search range.

Because of the long computation time and pointless sweeping of the whole search range, an improvement of the brute-force method was suggested. It was developed based on the branch-and-bound method, in which the candidate solutions with worse cost function value are rejected with all subsequent subsolutions. It was decided that, for draft synthesis, a similar approach could be adopted to search the range of the suspension dimensions. The flowchart of the improvement was presented in Figure 6. The initial search range was divided into two subsets. Solutions from the centre of the new search ranges were calculated and validated with a quality indicator. The quality indicator of “*lower range*” was compared with result obtained with solution from “*upper range*” and the worse result with the whole search range was rejected accordingly. The process was repeated till the size of search range was comparable with the values of step size presented in the brute-force method. The main advantage of the proposed improvement was the reduction of the considered solutions and a noticeable decrease in the computation time. However, the best solution may have been rejected with the whole search range. Nevertheless, it is suitable for problems where the cost function is not drastically variable, and the adjacent solutions are approximately similar.

## 4. Chameleon Swarm Algorithm Application

As it was proved in previous paragraphs, the standard methods of searching the demanded size require a full review within defined range variables, even if the next results are worse than the previously obtained. In most cases, this may result in unsatisfactory performance. The second presented algorithm reduces the number of required calculations by dividing the search ranges and discarding the halves based on single computation results. Despite reducing the computation time, the obtained solution may not be the best for the initial search range.

Because of the mentioned issues, a novel approach was applied to the synthesis problem. A new metaheuristic optimization algorithm, the Chameleon Swarm Algorithm, was chosen. The application of the metaheuristic algorithm warrants that any search range will not be discarded. The user also can define a maximum number of iterations regardless of the size of the optimization range.

The Chameleon Swarm Algorithm is a novel metaheuristic, bio-inspired algorithm. Braik proposed it in 2021, and it was inspired by chameleon hunting behaviour [22]. The unique ability of chameleons is their sight, which enables a viewing area of 360${}^{\circ }$ and may be focused on prey. The algorithm has five parameters that the user can modify. These are:

- number of iterations $I_{max}$,
- size of population *N*,
- control parameter σ,
- control parameter τ,
- control parameter ρ.

Although the algorithm was developed recently, there are papers describing the utilization of the algorithm in different disciplines. It has been successfully applied in electronic device design [32,33], economical power distribution [34], model identification of photovoltaic cells [35] and solid oxide fuel cells [36]. Braik provided results for benchmark functions and basic mechanical structures (e.g., bearings, springs, welded connection) optimization [22] However, the CSA has never been applied for the task similar to the described kinematic chain geometric synthesis, proving the novelty of the research topic. In comparison with the well-known Particle Swarm Optimizer (PSO) [37], the selected algorithm should ensure the stability of the optimization process without additional meta-optimization of the parameters. Even though the number of customizable parameters is similar, the ease of application with the default values can be noticed.

### 4.1. Initialization

The algorithm can be divided into segments that reproduce different stages of the hunting process. In the beginning, an initial population of chameleons is generated, in which every specimen denotes a possible solution to the optimization problem. Positions of the chameleons create *N* × *d* matrix of solutions, where *N* is the size of the population, and *d* is the number of searched values. In the specific problem of described type synthesis, we have d=4. Every specimen may be described with a vector (10):

(10) $$x_{i}^{n}=\left[x_{i}^{n,1},x_{i}^{n,2},x_{i}^{n,3},\ldots ,x_{i}^{n,d}\right]$$

where: *n* = 1, 2, 3, *…*, *N*—number of specimen in the population; *i*—number of iterations; *d*—number of searched values. The values of the initial population $x_{0}^{n}$ are randomly generated according to the Equation (11):

(11) $$x_{0}^{n,d}=l_{b_{d}}+v\times \left(u_{b_{d}}-l_{b_{d}}\right)$$

where: *d*—number of dimensions; *n*—number of specimen, $l_{b_{d}}$—lower bound of search range; $u_{b_{d}}$—upper bound of search range; *v*—randomly draw value from range [0,1]. Then the fitness function for every solution is calculated.

### 4.2. Prey Prospection

**Figure 7:**
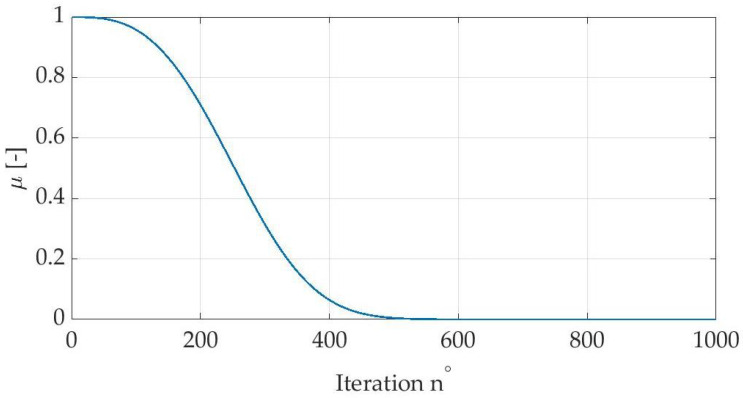
Change of the μ factor in subsequent iterations.

New solutions are obtained from the current solution by adding an improvement factor (12), calculated according to Equation (13) which describes position update for two possible situations. The first one represents global optimization and occurs when the randomly generated switch value is higher than Pp, which is the probability of noticing prey by the chameleon. Users may tune this parameter. However, based on the literature, it can be assumed Pp=0.1. The second situation represents local optimization and occurs when the drawn switch *s* is lower than Pp.

(12) $$x_{i+1}^{n,d}=x_{i}^{n,d}+\Delta_{x_{i}^{n,d}}$$

where: $x_{i+1}^{n,d}$—new solution; $x_{i}^{n,d}$—current solution; $\Delta_{x_{i}^{n,d}}$—improvement factor.

(13) $$\Delta_{x_{i}^{n,d}}=\{\begin{array}{c} \sigma \left(g_{i}^{n,d}-G_{i}^{d}\right)v_{1}+\tau \left(G_{i}^{d}-x_{i}^{n,d}\right)v_{2}\;s⩾P_{p}\\ \mu \left(\left(u_{b_{d}}-l_{b_{d}}\right)v_{3}+u_{b_{d}}\right)sgn\left(v_{4}-0.5\right)\;s<P_{p} \end{array}$$

where: τ,σ—the customizable CSA parameters; $u_{b_{d}},l_{b_{d}}$—upper and lower bound of search range; *sgn()*—signum function returning 1 or −1; $v_{1},v_{2},v_{3},v_{4}$—random numbers drawn in every iteration from range [0,1]; $g_{i}^{n,d}$—best solution obtained so far by the exact *n*th specimen; $G_{i}^{d}$—global best solution so far; *s*—randomly generated switch value; μ—step factor, decreasing in next iterations, defined with Equation (14) and presented in Figure 7.

(14) $$\mu =\gamma e^{{\left(-\kappa i/I_{max}\right)}^{\phi }}$$

where: γ,κ,ϕ—constant parameters with values: 1, 3.5 and 3 tuned by Braik empirically; *i*—number of current iteration; $I_{max}$—maximum number of iterations.

As shown in Figure 7, during initial iterations, the value of the step factor is the highest. It decreases gradually and remains marginal until the end of the optimization process. This means that at the beginning reach range of the chameleons is relatively large regardless of the *s* switch, and the optimization process is global. Then the μ factor allows only perceiving the nearest prey.

### 4.3. Rotation of Chameleons’ Eyes

The special ability of chameleons was also implemented in the algorithm. The $360^{\circ }$ eyes’ rotation enables noticing prey. It is assumed that hunting requires chameleons to update their position (move and rotate) to the prey location. This can be modeled with four step sequence (presented below).

1. Chameleon is moved to the centre of gravity (translation to the origin).
2. Based on the noticed prey position, the rotation matrix is created.
3. The chameleon is rotated accordingly at the origin.
4. The rotated chameleon is translated to the initial position.

**Figure 8:**
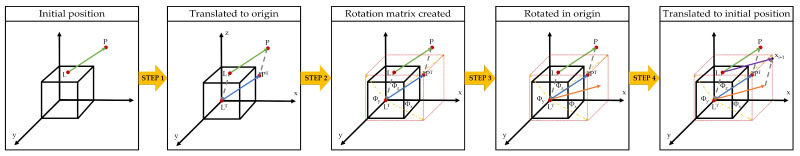
The rotation step of the CSA.

The presented steps can be observed in Figure 8. In the illustration, point L represents the chameleon, and point P represents the prey position. This sequence may be described with the below equations. The updated position of the rotated chameleon is defined as:

(15) $$x_{i+1}^{n}=xr_{i}^{n}+\bar{x_{i}^{n}}$$

where: $x_{i+1}^{n}$—position after rotation; $\bar{x_{i}^{n}}$—center position of the chameleon before rotation; $xr_{i}^{n}$—rotation in centered coordinates, obtained with Formula (16):

(16) $$xr_{i}^{n}=R\times xc_{i}^{n}$$

where: *R*—rotation matrix (17); $xc_{i}^{n}$—centering coordinates (18). The elements of the Formula (16) may be calculated using below dependencies:

(17) $$R=rot(\Phi ,\vec{t_{1}},\vec{t_{2}})$$

where: $\vec{t_{1}},\vec{t_{2}}$—orthonormal d×1 vectors; Φ—rotation angle of the chameleon (calculated with Equation (19)), *rot*—rotation matrices in the *x, y* axes (Formulas (20) and (21)).

(18) $$xc_{i}^{n}=x_{i}^{n}-\bar{x_{i}^{n}}$$

where: $x_{i}^{n}$—initial position of the chameleon. The rotation angle of the chameleon in the CSA is defined with Equation (19):

(19) $$\Phi =vsgn\left(v_{4}-0.5\right)\times 180^{\circ }.$$

The rotation matrices along *x* and *y* axes are represented with the below values:

(20) $$rot_{x}=\left[\begin{array}{ccc} 1 & 0 & 0\\ 0 & \cos\phi & -\sin\phi \\ 0 & \sin\phi & \cos\phi \end{array}\right]$$

(21) $$rot_{y}=\left[\begin{array}{ccc} \cos\Phi & 0 & \sin\Phi \\ 0 & 1 & 0\\ -\sin\Phi & 0 & \cos\Phi \end{array}\right].$$

The described rotation process occurs for every specimen in the population, regardless of the searching prey stage.

### 4.4. Hunting

The final step of the algorithm represents the chameleon attacking the nearest prey with a long tongue. The position of prey reached with the tongue represents the solution of the CSA iteration and can be computed using Equation (22):

(22) $$x_{i+1}^{n,d}=x_{i}^{n,d}+\left({\left(p_{i}^{n,d}\right)}^{2}-{\left(p_{i-1}^{n,d}\right)}^{2}\right)/\left(2a\right)$$

where: $p_{i}^{n,d},p_{i-1}^{n,d}$—speed of the tongue in the current and the previous iteration (24); *a*—the acceleration of chameleon’s tongue defined with below Formula (23):

(23) $$a=2590\times (1-e^{-log(i)})$$

where: *i*—number of iteration; 2590 m/s${}^{2}$ is the maximum acceleration of the chameleon’s tongue [38].

**Figure 9:**
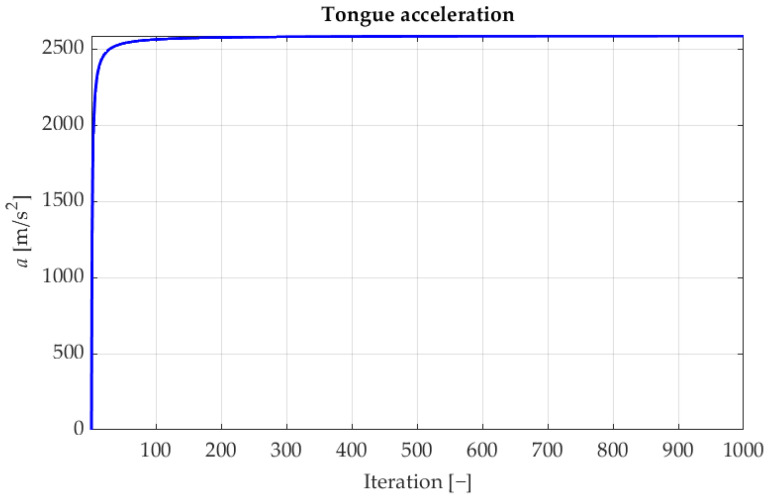
The *a* parameter in the following iterations of the CSA algorithm.

As shown in Figure 9, the acceleration increase occurs in the initial iterations and then remains steady with only a subtle growth trend. The last missing element of the algorithm is the calculated speed of the tongue, which is defined with Equation (24)

(24) $$p_{i+1}^{n,d}=\omega p_{i}^{n,d}+c_{1}\left(G_{i}^{d}-x_{i}^{n,d}\right)v_{1}+c_{2}\left(g_{i}^{n,d}-x_{i}^{n,d}\right)v_{2}$$

where: $c_{1},c_{2}$—constant value adjusting influence of global best and *n*th specimen solution; ω—inertia weight, defined with Equation (25):

(25) $$\omega ={\left(1-i/I_{max}\right)}^{\rho \sqrt{i/I_{max}}}$$

where: ρ—exploitation capacity parameter.

**Figure 10:**
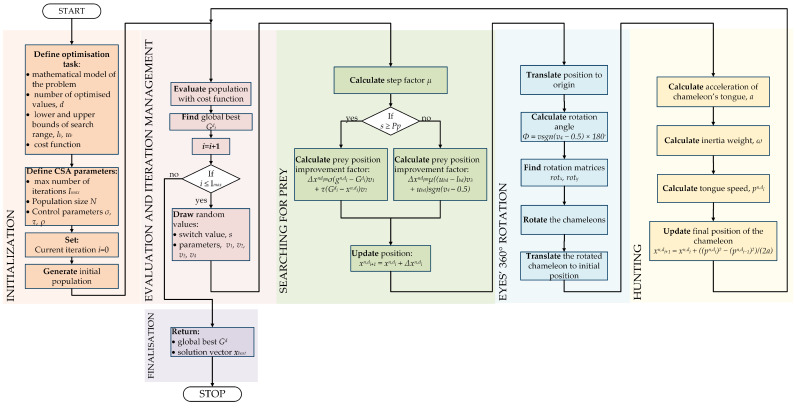
Flowchart of the CSA optimization.

After the four steps of the CSA are computed, the population is analyzed and validated through the cost function. The best solution is compared with the global best, and the global best is updated if improvement is obtained. The process is executed until a maximum number of iterations is reached (Figure 10). Because of the novelty of the approach, the stopping criteria were not employed in the conducted tests. However, for further application, it is suggested to check if the improvement is reached within the last 10 iterations. If the cost function value is not decreasing, the optimization process can be interrupted.

### 4.5. Application

The most important part of the approach with the application of the optimization algorithm was the necessity of defining the cost function. In every iteration, the value of the cost function was calculated and compared with the global best. The optimal solution of the optimization problem was characterized by the minimal value of the cost function.

In the considered application, the most important property of the wheel trajectory was to be possibly linear and perpendicular to the base surface. For this reason, the error along *x* axis was more significant rather than the less important errors in *y* axis, resulting in the robot clearance. This resulted in defining weights of the errors in the ratio 4:1, in a consequence, the cost function was given with Equation (26):

(26) $$F_{cost}=\frac{4\times E_{x}^{2}+E_{y}^{2}}{5}$$

where $E_{x},E_{y}$ is the sum of all distances along the *x* and *y* axes. The type synthesis problem was defined as a function of only four variables, and all transformations from Section 2 depend only on optimized values. After such conversion the solution vector (10) in the specific problem took the form (27):

(27) $$x_{i}^{n}=\left[b_{i}^{n},c_{i}^{n},x_{D_{i}}^{n},y_{D_{i}}^{n}\right]$$

where: $b_{i}^{n},c_{i}^{n}$—lengths of appropriate bars; $x_{D_{i}}^{n},y_{D_{i}}^{n}$—position of the fixing point D. Missing parameter β was calculated on the basis of rockers dimensions and initial point of the desired trajectory. The search range for the optimization task was limited by the previously set boundaries (Table 1).

**Figure 11:**
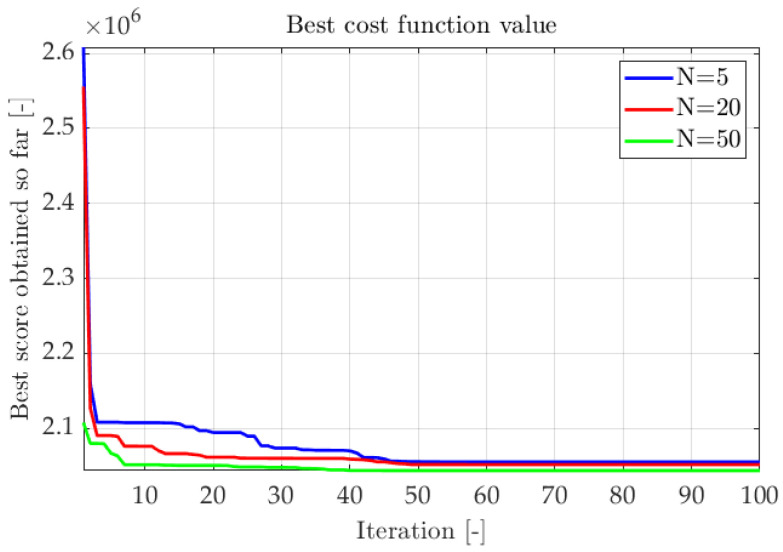
Changes of fitness function - different numbers of specimens in population.

**Figure 12:**
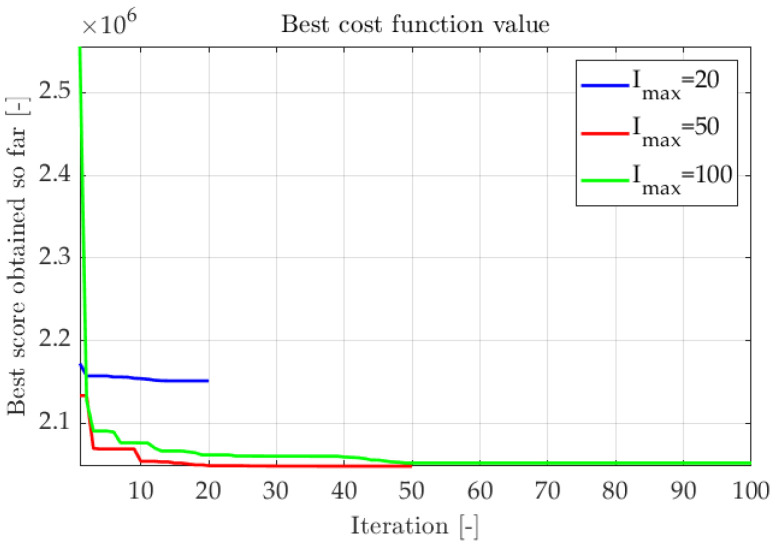
The fitness function values for different numbers of iterations.

Multiple optimization sequences were conducted to adapt adequate algorithm parameters. The evaluation factor was the improvement of the cost function. In Figure 11 and Figure 12 fitness functions from subsequent trials were compiled. The first test aimed to determine the impact of population size on algorithm efficiency. The number of iterations $I_{max}$ was set to 100, and the customizable parameters remained default values:

$$1^{st}test:\begin{array}{cc} N & variable\\ I_{max} & 100\\ \tau & 2.0\\ \sigma & 2.0\\ \rho & 1.0 \end{array}$$

$$2^{nd}test:\begin{array}{cc} N & 20\\ I_{max} & variable\\ \tau & 2.0\\ \sigma & 2.0\\ \rho & 1.0 \end{array}.$$

As shown in Figure 11 and Figure 12, most of the tests finished successfully, reaching similar cost function values. The higher the number of specimens in an iteration, the quicker optimal results are obtained. The number of iterations should be adapted to the problem (Figure 12). If the number of iterations is too small, the optimization can be inefficient. However, if the number of iterations is too high, the improvement is marginal after reaching a near-optimal solution. Analysis of the actual error value can be a guide for correct stopping criteria.

**Figure 13:**
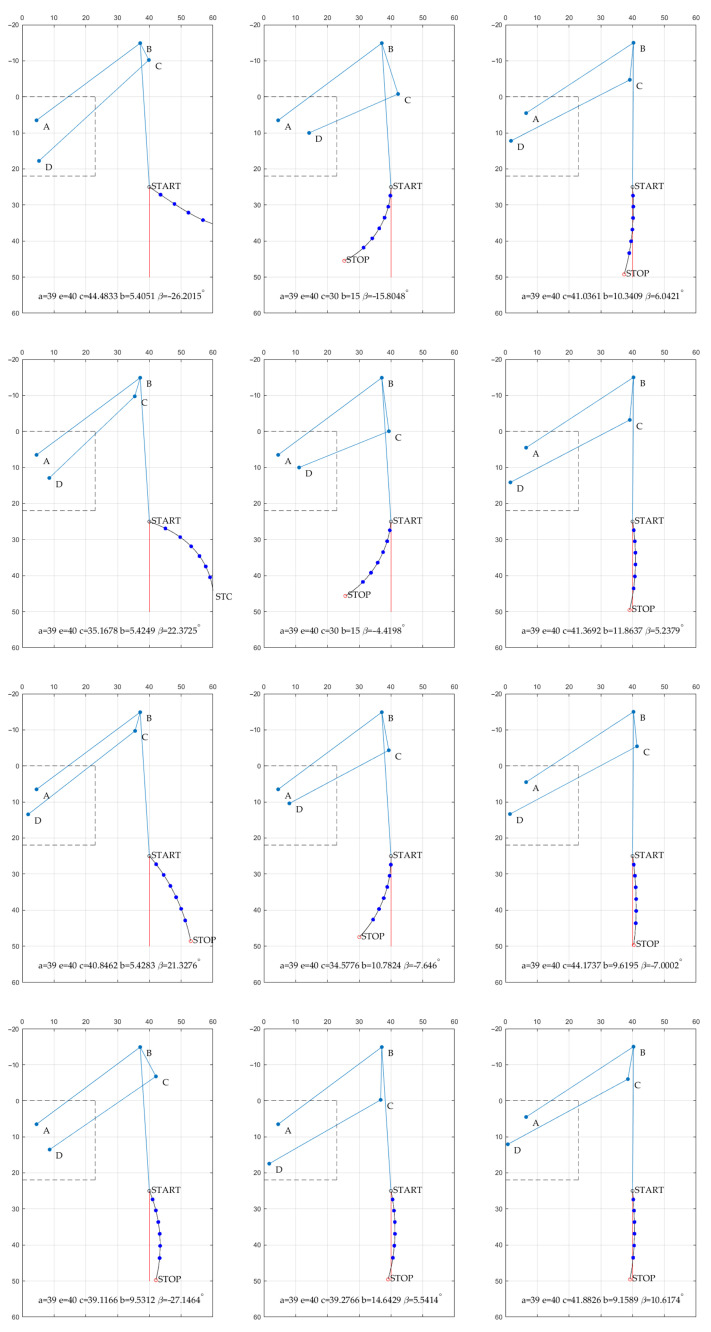
The examples of the trajectories achieved in the subsequent iterations of the optimization process (blue lines—linkage structure, red line—desired trajectory, dark blue doted line—obtained trajectory).

Multiple trajectories were validated, and the chosen examples can be observed in Figure 13. It can be noticed that the β angle may be both negative or positive. However, the solutions with negative values were more desirable. In the indicated situation, the overlapping parts are eliminated, and it is easier to fix the construction. For the prototype construction, the obtained values were rounded off to the nearest full dimension in centimeters. The rounded values are presented in Table 2. The correctness of the optimization was confirmed with the preliminary test of the Grey Wolf Optimizer (GWO) algorithm [39], that will be considered in further research activities. The results obtained with two different algorithms were very similar, and the difference was negligible for the prototype application as they did not exceed the machining tolerances.

Based on the optimization results (Table 2), a 3D model of the structure was created. The elements of the required lengths were modeled as simple flat bars. The CD bar was also modeled as a constant length element because it was assumed at the beginning of the synthesis that only a single drive was required to follow the desired trajectory. The variable length of this element was required by the stepping and not the levelling function of the suspension. The correlations between the elements were defined, and the propulsion of the fixing point was performed. All joints were simulated as rotational kinematic pairs. The BCM angle (β) was constrained as a constant placement between two elements. The robot platform was replaced with a cuboid which was fixed to the environment (it was not able to move). The mounting points were attached in the designated positions of the cuboid. The wheel was attached at the end of the kinematic chain to make the model attractive; however, the wheel was not propelled.

**Figure 14:**
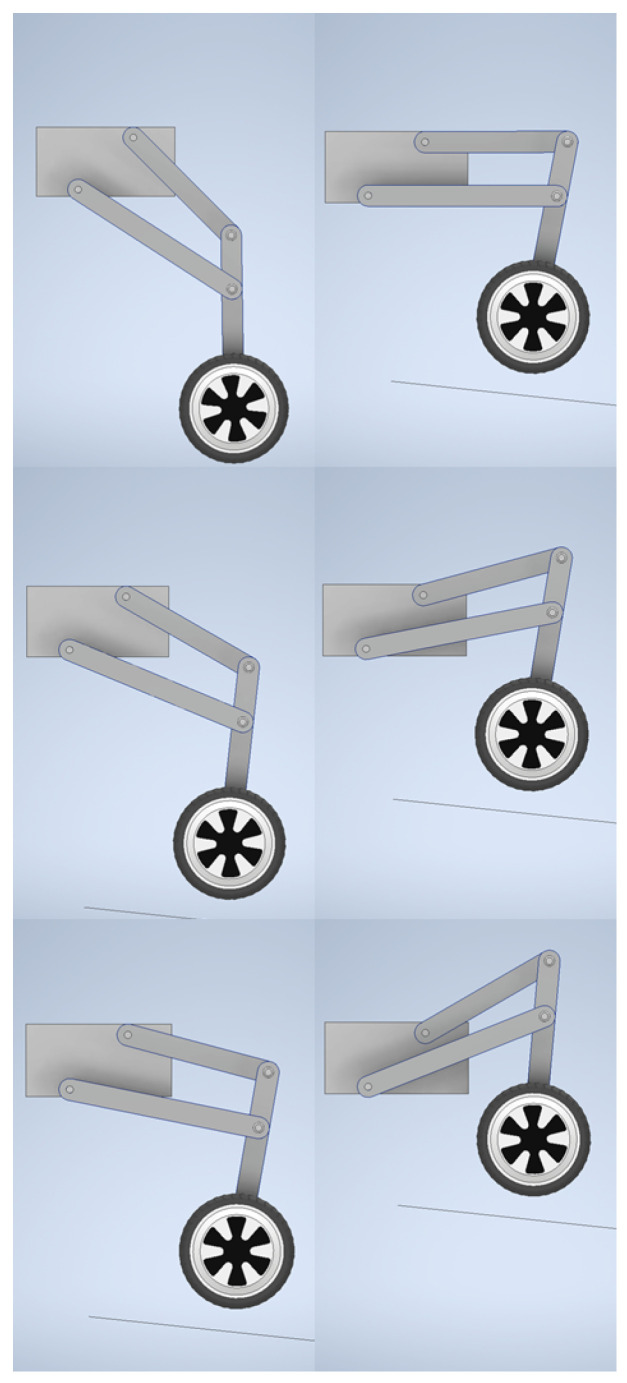
The simulation of the leg movement for the rocker angle (α range from −45${}^{\circ }$ to 30${}^{\circ }$).

The model was animated by applying the variable angle of AB link from the range $-45^{\circ }$ to $30^{\circ }$ to the ground surface. This was achieved with the animate constraint function of Autodesk Inventor software. The rotations in the remaining joints were calculated by the software without user interference. The obtained trajectory of the wheel (that can be noted in next positions of the wheel presented in Figure 14 confirmed that the results of the optimization could be used in the preparation of the prototype.

## 5. Experimental Results and Walking Principles—Overview of Control System

The research was conducted not only to compare different methods but also to collect the data to construct a real prototype of a wheel-legged robot. The dimensions obtained during the synthesis were used to prepare a prototype of the robot’s leg. The *c* rocker was replaced with an adjustable linear actuator. The HIWIN LAS150 electrical actuator was chosen. For the basic control, the linear actuator can be blocked at the desired length, allowing the wheel axis to reproduce the previously mentioned trajectory with only a single drive. The actuator stroke is 150 mm. However, it was decided to reduce the effective stroke by the software in order to obtain symmetrical movement from the initial length.

**Figure 15:**
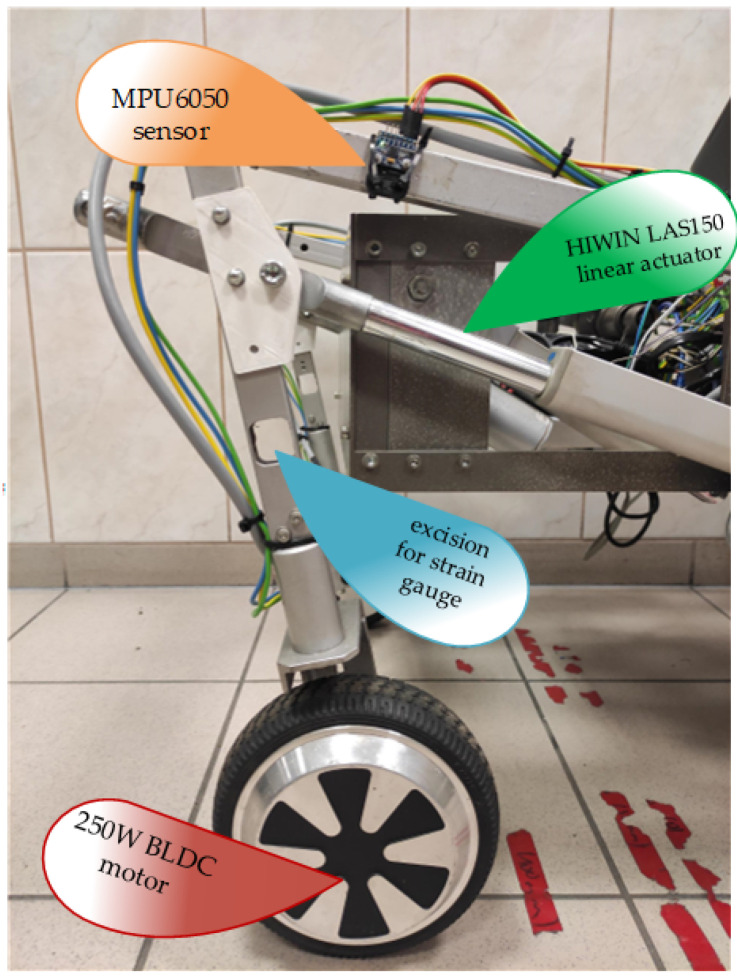
The non-turnable rear leg.

The angle of AB rocker was measured with low-cost MEMS (*MicroElectroMechanical System*) accelerometer and gyroscope MPU6050 (Figure 15). This allowed the control system to work with feedback from the linear elongation of the linear levelling actuator and the angular rotation of the rocker. The doubled measurement was planned to be utilized for employing an artificial neural network to increase the precision of the suspension configuration [40]. Another advantage of the redundant sensor was the ability to continue the robot’s operation under the gyroscope or the rotary encoder malfunction. It prevented unsuspected downtime and maintenance, which were unwanted in autonomous and remote vehicles [41].

The linear displacement of the actuator is calculated based on the rotary encoder indication. The encoder is directly attached to the shaft of the lead screw of the mechanism.

**Figure 16:**
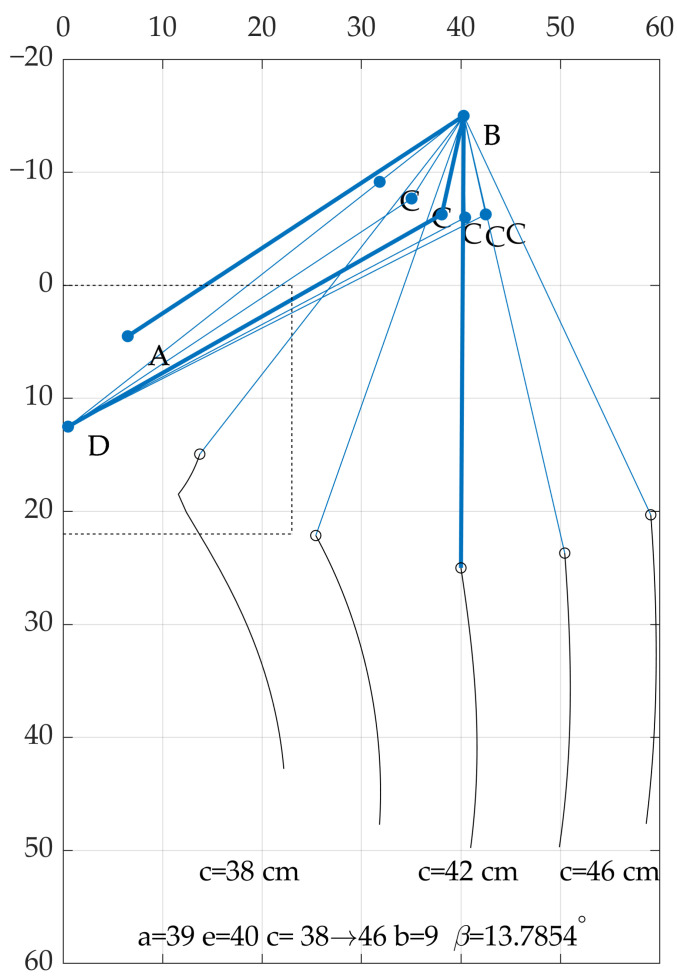
The influence of *c* length on position of robot’s wheel (blue lines—linkage structure, black curves—obtained trajectories).

The front robot leg is equipped with four motors, and each is responsible for a different degree of freedom. Because of the required dynamics, especially high torque at a standstill and low speeds, the levelling and walking are obtained with electric linear actuators. The control structure was implemented in the dispersed microcontroller system. The master controller, which was also responsible for simultaneous visual environmental data acquisition and localization, calculates the desired speeds or positions of all drives. This part was implemented in a terminal PC with installed ROS (*Robotic Operating System*). The environmental data required for SLAM (*Simultaneous Localization and Mapping*) were gathered with a Microsoft Kinect RGBD camera. The extension of the vision system with an IR depth camera enhanced the efficiency of obstacle detection. The master controller was responsible for classifying the obstacle—whether it could be driven around or it was required to be stepped over. The walking technique required inverse kinematics calculation. On the basis of desired position of the M point the AB rocker (α) angle and *c* element length were computed. While the movement along the x-axis was not taken into account, the problem was linear because of the described structure of the suspension. While both x and y displacement were required, the inverse kinematics required more complex transformation because the change of *c* length not only affected x movement but also resulted in the movement along the y-axis. The influence of *c* length is presented in Figure 16.

As it can be concluded from Figure 16, the accuracy of *c* length control is very crucial for correct trajectory. For this reason, in further research, additional sensors will be validated. In current form, the *c* length was calculated basing on the rotary encoder readings.

**Figure 17:**
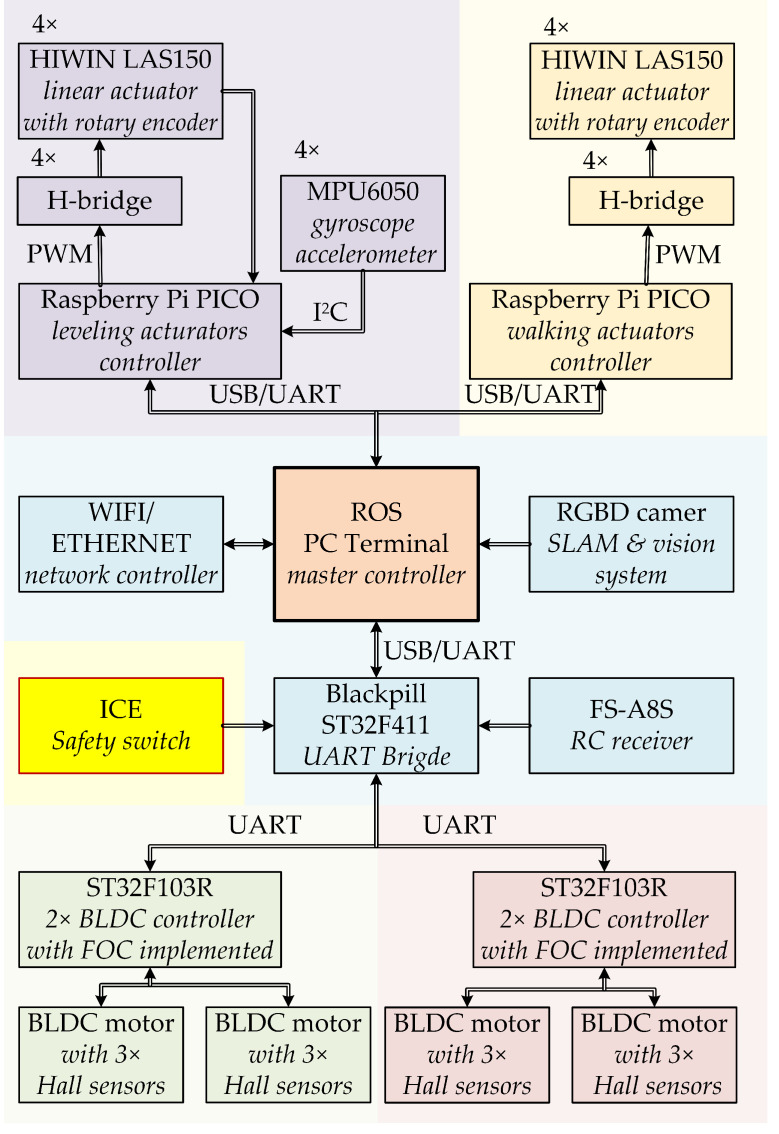
The scheme of the control structure.

The length was directly computed by the microcontroller responsible for levelling and walking. The linear actuators’ control systems were implemented on two Raspberry Pi PICO boards. The scheme of the control system is shown in Figure 17.

## 6. Conclusions and Future Work

The current trends in robotic platforms, the requirements and the disadvantages of different suspension constructions were reviewed. The efficient hybrid wheel-legged construction of the inspection robot was proposed. The mathematical model of the structure was provided.

Three methods of type synthesis were presented. The standard brute-force method was compared with the proposed improvement based on the branch and bound algorithm. The novel synthesis approach with a metaheuristic nature-inspired optimization algorithm was described in detail. The Chameleon Swarm Algorithm was described, and the required application steps were presented. The numeric problem simulation results were provided, and the experimental verification was shown. The prototype was briefly described, and the main parts of the control system, including applied sensors and microcontrollers were indicated.

The presented results confirmed that the CSA optimizer is an efficient robot suspension system type synthesis tool. In comparison with standard methods, the search range is smartly swept. Instead of a naive brute force method, the next iterations are based on cost function value. The proposed algorithm warrants complete search range verification. The obtained solution was successfully tested in 3D modelling software and a real prototype.

The contribution of the paper was a presentation of the application of the novel metaheuristic algorithm in the synthesis of the suspension for the mobile robot for mining infrastructure inspection. The requirements, trends and problems in the synthesis of mobile robots, especially the complex suspension systems, were described. Four linkage structure was described and used as a model and optimization task in the further part of the paper. The successful application of the Chameleon Swarm Algorithm is provided. Simulation and experimental tests both confirmed the results. Finally, the general idea of the control structure of the drives applied in a robot, including sensors, microcontrollers and power electronics, was given.

The presented design was confirmed with the assembly of the functional prototype in its preliminary form. The improvement of the robot will be the main topic of further research. The control system will be enhanced with the vision system enabling obstacle detection and the autonomous operation of the robot. Additionally, the parameters of the speed controllers applied to the drives will be tuned with different optimization algorithms. For comparison purposes, the application of the different algorithms (the GWO) for the suspension structure is considered. It is also planned to develop a more complex mathematical model of the leg that may be used for internal stress analysis. Further research concerning the prototype will be focused on the experimental evaluation of the robot. Because of the rules defined by law, the robot in its current form, without additional costly certification, can be employed, for example, in copper mines and warehouses. The movement and levelling performances are planned to be examined, and the measurement of the vibrations’ influence on the transported goods is also considered, and for this purpose, an additional sensory system will be developed and employed in the prototype.

## Figures and Tables

**Table 1:** Input parameters for the brute-force synthesis.

Parameter	Range		Constant Value
	Lower Bound	Upper Bound	
a			39 cm
*b*	5 cm	15 cm	
*c*	30 cm	45 cm	
e			40 cm
$X_{A}$			6.5 cm
$Y_{A}$			4.5 cm
$X_{D}$	0 cm	10 cm	
$Y_{D}$	10 cm	20 cm	
$\Delta_{b}$			0.5 cm
$\Delta_{c}$			1 cm
$\Delta_{X_{D}},\Delta_{Y_{D}}$			0.5 cm

**Table 2:** Dimensions of the suspension applied in the prototype of the robot.

Parameter	*b*	*c*	β	$X_{D}$	$Y_{D}$
**Value**	9 cm	42 cm	$11^{\circ }$	0.5 cm	12 cm

## Data Availability

Not applicable.

## Abbreviations

The following abbreviations are used in this manuscript:

SI	Swarm Intelligence
CSA	Chameleon Swarm Algorithm
PSO	Particle Swarm Optimizer
GWO	Grey Wolf Optimizer
MEMS	MicroElectroMechanical System
ROS	Robotic Operating System
SLAM	Simultaneous Localization and Mapping
BLDC	Brushless DC (motor)
FOC	Field Oriented Control
RC	Remote Controller
